# Intra- and interobserver reliability of glenoid fracture classifications by Ideberg, Euler and AO

**DOI:** 10.1186/s12891-018-2016-8

**Published:** 2018-03-27

**Authors:** F. Gilbert, L. Eden, R. Meffert, F. Konietschke, J. Lotz, L. Bauer, W. Staab

**Affiliations:** 1 Department of Trauma, Hand, Plastic and Reconstructive Surgery, Julius-Maximilians-University of Würzburg, Würzburg, Germany; 2 Department of Mathematical Sciences, The University of Texas at Dallas, Richardson, TX USA; 3 Institute for Diagnostic and Interventional Radiology, University Medical Center Göttingen, Göttingen, Germany

**Keywords:** Scapula, Glenoid, Fracture, Classification, Diagnosis, Reliability, Comparison

## Abstract

**Background:**

Representing 3%–5% of shoulder girdle injuries scapula fractures are rare. Furthermore, approximately 1% of scapula fractures are intraarticularfractures of the glenoid fossa. Because of uncertain fracture morphology and limited experience, the treatment of glenoid fossa fractures is difficult. The glenoid fracture classification by Ideberg (1984) and Euler (1996) is still commonly used in literature. In 2013 a new glenoid fracture classification was introduced by the AO. The purpose of this study was to examine the new AO classification in clinical practice in comparison with the classifications by Ideberg and Euler.

**Methods:**

In total CT images of 84 patients with glenoid fossa fractures from 2005 to 2018 were included. Parasagittal, paracoronary and axial reconstructions were examined according to the classifications of Ideberg, Euler and the AO by 3 investigators (orthopedic surgeon, radiologist, student of medicine) at three individual time settings. Inter- and intraobserver reliability of the three classification systems were ascertained by computing Inter- and Intraclass (ICCs) correlation coefficients using Spearman’s rank correlation coefficient, 95%-confidence intervals as well as F-tests for correlation coefficients.

**Results:**

Inter- and intraobserver reliability for the AO classification showed a perspicuous coherence (*R* = 0.74 and *R* = 0.79). Low to moderate intraobserver reliability for Ideberg (*R* = 0.46) and Euler classification (*R* = 0.41) was found. Furthermore, data show a low Interobserver reliability for both Ideberg and Euler classification (*R* < 0.2). Both the Inter- and Intraclass reliability using AO is significantly higher than those using Ideberg and Euler (*p* < 0.05). Using the new AO classification, it was possible to find a proper class for every glenoid fossa fracture. On average, according to Euler classification 10 of 84 fractures were not classifiable whereas to Ideberg classification 21 of 84 fractures were not classifiable.

**Conclusion:**

The new AO classification system introduced 2013 facilitates reliable grading of glenoid fossa fractures with high inter- and intraobserver reliability in 84 patients using CT images. It should possibly be applied in order to enable a valid, reliable and consistent academic description of glenoid fossa fractures. The established classifications by Euler and Ideberg are not capable of providing a similar reliability.

## Background

Comprising 3%–5% of shoulder girdle injuries scapula fractures are uncommon. Scapula fractures are uncommon but make up 3–5% of all shoulder girdle injuries. Moreover, intraarticular glenoid fossa fractures represent only 1% of scapula fractures [1]. However, fractures of the scapula may not only lead to serious pain but also affect regular function of the shoulder girdle leading to scapulothoracic dyskinesis, malunion, nonunion, rotator cuff dysfunction or impingement, respectively [1–3].

Due to different injury mechanisms, glenoid avulsions, rim fractures and fossa fractures should be distinguished from each other. Anterior dislocations of the shoulder caused by low energy- or sports trauma are generally responsible for glenoid avulsions and rim fractures [3, 4]. By contrary, high energy blunt-force mechanisms are accountable for glenoid fossa fractures [2, 5]. Thus, glenoid fossa fractures are frequently found with concomitant injuries to chest, head, brachial plexus and humerus. According to Voleti 80% to 95% of glenoid fossa fractures are associated with additional injuries, whereas to van Oostveen up to 60% [1, 6].

The treatment of scapula fractures has changed from preferably conservative to operative. Recent reviews of Zlowodzki and Lantry have shown that operative treatment was used for 80% of all glenoid fractures with good to excellent results in 82% of the cases [7, 8]. In general, indication for surgery depends on instability, degree of dislocation and articular surface fragment size [6]. On the one hand, there is a high number of publications about glenoid fracture treatment. On the other hand, comparing these publications is challenging because many different classification systems for description of scapula fractures were used.

In summary, plenty of classification systems for glenoid fractures exist. One of the most commonly used classification is the one by Ideberg [9, 10]. Based on standard radiographs solely, Ideberg originally classified intraarticular fractures into 5 main types of fracture patterns. Later his classification was altered by Goss and Mayo [2, 11]. Additionally Goss showed that Ideberg’s classification has got no prognostic value [2].

Another popular scapular fracture classification especially in German literature is Euler’s classification [12]. In the subdivision for glenoid fractures Euler distinguishes between 6 different types of fracture patterns. Moreover, types of fracture patterns can be combined.

In 2013, the Orthopaedic Trauma Association (OTA) and the Arbeitsgemeinschaft für Osteosynthesefragen (AO) came up with their new OTA/AO classification of scapular fractures based on the analysis of 45 CT scans [13]. This classification divides fractures of the glenoid in 11 possible fracture patterns dividing the glenoid fossa in four quadrants. Diverse opinions about the new AO classification are found in the current literature. For example, Bartoníček et al. question its practical relevance and claim it includes a hypothetical fracture [14]. By contrary, ter Meulen et al. conclude that the new AO classification allows adequate characterization and discrimination of glenoid fracture patterns focusing on the number of fragments, fragmented articular surface area and their relation to the type of injury [15].

Hence, the purpose of this study was to examine the new AO classification in clinical practice in comparison to the classifications by Ideberg and Euler. We hypothesized that applying the new AO classification system leads to superior inter- and intraobserver correlation than using the classifications by Ideberg or Euler.

## Methods

**Fig. 1:**
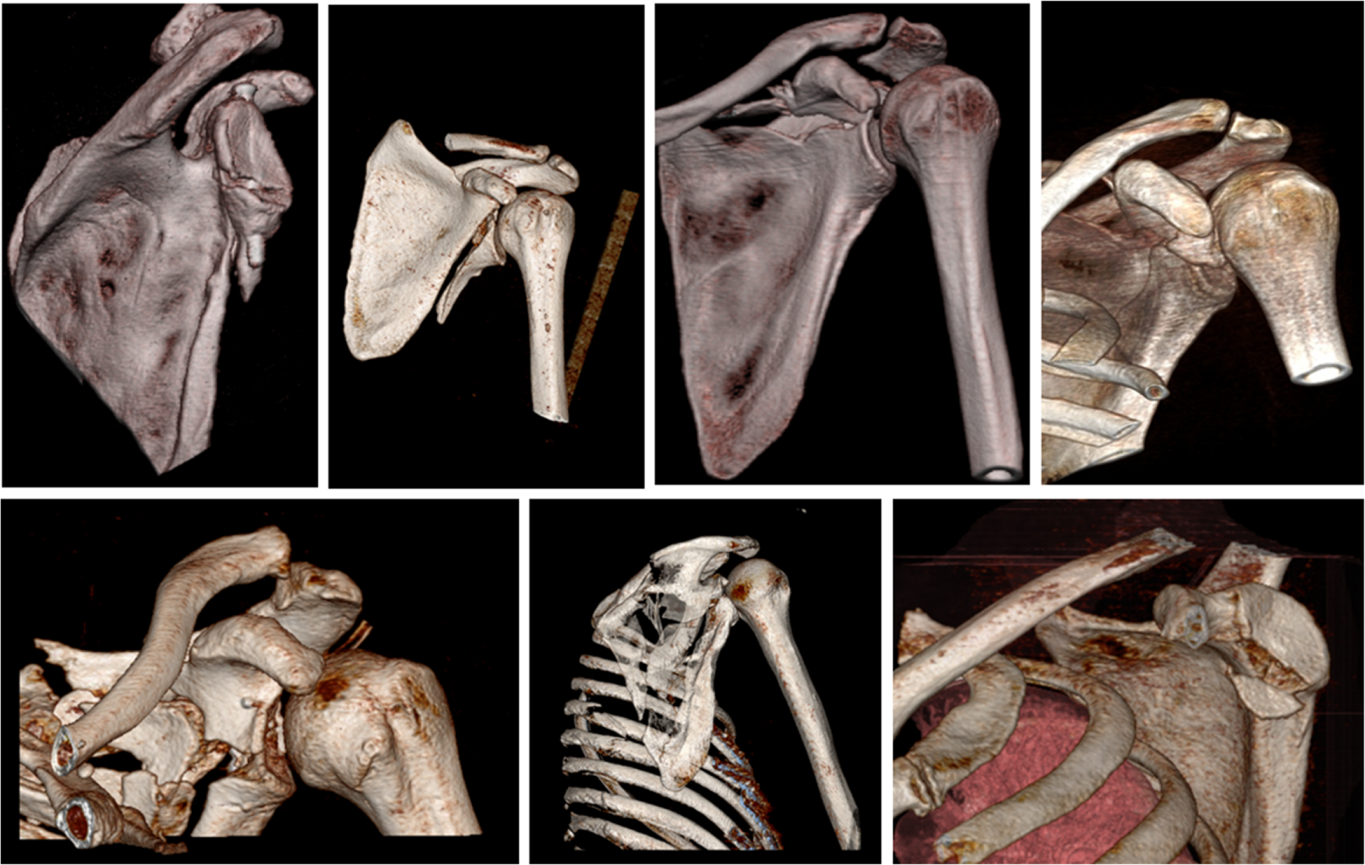
Examples of glenoid fractures and in combination with the scapular body and neck

A retrospective study was performed in patients who were diagnosed with fractures of the glenoid fossa from 2007 to 2018. A total of 84 cases were found. 73 males and 11 females with a median age of 34 years (range 16–71). The dominant hand was injured in 50 patients. Every patient had posteroanterior(PA), 30° oblique pronation and lateral radiographs following injury and then had a CT scan with 3D reconstruction if further evaluation was required for the confirmation of diagnoses. The 64-channel volume CT (Light speed® VCT XT, GE Healthcare, Milwaukee, USA) was used with the following protocol; slice thickness of 2 mm, tube voltage of 120 kV, tube current of 1.10 mAs, helical type scan, 0.5 s of rotation, and reconstruction with bone kernel. We classified glenoid fractures according to the classifications by Ideberg, Euler and AO based (Tables 1, 2 and 3) on 3D CT images (Fig. 1).

**Table 1:** Ideberg classification of glenoid fossa fractures

Type Ia	Anterior rim fracture
Type Ib	Posterior rim fracture
Type II	Fracture line through glenoid fossa exiting scapula laterally
Type III	Fracture line through glenoid fossa exiting scapula superiorly
Type IV	Fracture line through glenoid fossa exiting scapula medially
Type Va	Combination of types II and IV
Type Vb	Combination of types III and IV
Type Vc	Combination of types II, III, and IV
Type VI	Severe comminution

**Table 2:** Euler and Rüedi classification for scapular fractures

A Fractures of the body of scapula	Isolated or multifragmentary	
B Fractures of the process	B1 spine B2 coracoid B3 acromion	
C Fractures of scapular neck	C1 anatomical neck C2 surgical neck C3 surgical neck with	a. fracture clavicle and acromion b. torn CC and CA ligaments
D Articular fractures	D1 glenoid rim D2 glenoid fossa with D3 scapula neck and body fracture	a. inferior glenoid fragment b. horizontal split of scapula c. coracoglenoid block formation d. comminuted fractures
E Fracture combination	with humeral head fractures

**Table 3:** AO/OTA classification (incomplete)

Scapula, extra-articular (not glenoid) (14-A)	A1: Acromion A2. Coracoid A3. Body	
Partial articular (glenoid) (14-B)	B1 Anterior rim B2 Posterior rim B3 Inferior Rim	1.1 Anterior rim, noncomminuted 1.2 Anterior rim, comminuted 2.1 Posterior rim, noncomminuted 2.2 Posterior rim, comminuted 3.1 Inferior rim, noncomminuted 3.2 Inferior rim, comminuted
Total articular (glenoid) (14-C)	C1 Extra-articular glenoid neck C2 Intra-articular with neck C3 Intra-articular with body	1.1 noncomminuted 1.2 comminuted 2.1 Intra-articular with neck, articular noncomminuted, neck noncomminuted 2.2 Intra-articular with neck 2.3 comminuted, articular noncomminuted Intra-articular with glenoid neck, articular comminuted

An orthopaedic surgeon with 10 years of experience, a consultant radiologist with 9 years of experience, and a 4th-year medical student who was trained for two months prior to the study, evaluated 84 cases of glenoid fractures three times during a 9-week interval using the classification systems by Ideberg, Euler and the AO based on parasagittal, paracoronary and axial CT scans of the shoulder. All observers were blinded to the treatment. To determine the intra- and interobserver reliability of the classifications, we conducted Spearman’s rank correlation and 95% confidence intervals.

### Statistics

Data was analyzed by computing inter-and intraclass observer reliability measures (Spearman’s rank correlation coefficient) as well as 95% confidence intervals. Reliability is targeted with an ICC ≥0.8. Hypotheses in the differences among the reliability measures were tested using approximate F-tests for correlation coefficients as well as Fisher’s test. Results were interpreted as significant if p < 0.05 [16]. .All computations were conducted using the freely available statistical computing environment R version 3.0.1 software (R Foundation for Statistical Computing, Vienna, Austria; http://www.r-project.org) using the psych package [17]. This retrospective study was approved by the institutional review board at our hospital.

## Results

The interobserver and intraobserver reliability of the new AO classification showed a notable coherence (*R* = 0.74; 95% CI = 0.64–0.91 and *R* = 0.79; 95% CI = 0.68–0.84, respectively). We found a low to moderate correlation for intraobserver reliability for both Ideberg (*R* = 0.46; 95% CI = − 0.16–0.95) and Euler classification (*R* = 0.41; 95% CI = 0.02–0.87). Interobserver reliability for Ideberg and Euler classification showed no correlation (*R* < 0.2). The AO classification has a significantly higher reliability compared to the two competitors (*p* < 0.05). Furthermore, the confidence intervals do not overlap. Inter- and intraobserver reliability of the AO classification was higher than those of the classifications by Ideberg and Euler. Using new AO classification, every glenoid fossa fracture was classifiable. On average, according to Euler classification 10 of 84 fractures were not classifiable whereas in the Ideberg classification 21 of 84 fractures were not classifiable.

## Discussion

The findings of our study demonstrates that the new AO classification obtains a higher inter- and intraobserver reliability (*R* = 0.74 and *R* = 0.79) compared to Ideberg (*R* = 0.46) and Euler classifications (*R* = 0.41). This result is supported by the discovery that making use of the new AO classification, the observers had no problems finding a suitable class for every fracture of the glenoid fossa. By contrast, the observers were not able to classify every glenoid fracture according to the classifications of Ideberg (21 of 84) and Euler (10 of 84). Therefore, the new AO classification from 2013 may facilitate the classification of glenoid fractures compared to Ideberg and Euler classification.

Different opinions about the new AO classification exist in recent literature. Bartoníček et al. not only doubted the practical relevance of the AO classification, and presented a new classification themselves [14]. Our study shows that in contrast to other established classifications the AO classification provides satisfying results regarding inter- and intraobserver reliability. The practical relevance of a classification should be developed based on high reliability from the view point of furthering communication among orthopedic surgeons and researchers, and developing appropriate treatment plans by using a common classification system.

Ter Meulen et al. came to the result that the new AO classification enables appropriate distinction of glenoid fractures examining 3D CT models of 53 fractures [15]. They found a significant variation of articular surface area and number of fracture fragments among the different classes of the AO classification. This finding may be a possible explanation for the high inter- and intraobserver reliability of the AO classification found in our study. Additionally, ter Meulen et al. observed a significant relation between high- vs low-energy trauma and the fragmented surface area and high- vs low-energy trauma and the number of fragments, which supports a probable clinical relevance of the AO classification. These findings were supported by Neuhaus et al. found a proportion of rater agreement of 81% between 135 orthopedic surgeons in a web-based survey with 35 scapula fractures for the new AO/OTA classification, which is conclusive to our findings [18].

Our study includes several limitations. Having only a small sample size of 84 cases might be too limited in order to highly recommend the superiority of the new AO classification compared with Ideberg and Euler classification. Our study lacks a clinical and radiological follow-up. Therefore, the clinical impact or importance is difficult to assess. Furthermore, there might be a bias of the reliability test results because every hospital uses a different protocol of CT. Finally, not evaluating the time spent during the classification of injury, information about the immediacy of the classifications cannot be presented. However, this is the first study examining inter- and intraobserver reliability of 3 different classification systems (Ideberg, Euler, AO) with the aim of comparing them in one study. Using three observers working in different disciplines and with different states of knowledge, this study may allow an assumption about the convenience of the mentioned classification systems. Further studies are needed not only to prove the reliability of the AO classification but also to examine its clinical relevance.

## Conclusions

The 2013 newly introduced AO classification system allows reliable grading of glenoid fossa fractures with high inter- and intraobserver reliability in 84 patients using CT images. It should possibly be applied in order to enable a valid, reliable and consistent academic description of glenoid fossa fractures. The established classifications by Euler and Ideberg are not capable of providing a similar reliability.

## Abbreviations

AO: “Arbeitgsgemeinschaft Osteosynthesefragen”
CT: Computed tomography
ICC: Inter- and Intraclass correlation
kV: Kilovolt
mAs: Miliampere-seconds
mm: Millimeter
OTA: Orthopaedic Trauma Association
